# The one-dose schedule opens the door to rapid scale-up of HPV vaccination

**DOI:** 10.1186/s12916-023-03097-x

**Published:** 2023-10-09

**Authors:** Zhuoru Zou, Lei Zhang

**Affiliations:** 1 China-Australia Joint Research Centre for Infectious Diseases, School of Public Health, Xi’an Jiaotong University Health Science Centre, Xi’an, Shaanxi China; 2 Melbourne Sexual Health Centre, Alfred Health, Melbourne, Australia; 3 Central Clinical School, Faculty of Medicine, Monash University, Melbourne, Australia

**Keywords:** HPV vaccination, One-dose schedule, Modeling, Cost-effectiveness

## Background

In 2020, the World Health Organization launched a global strategy to accelerate the elimination of cervical cancer as a public health concern, with achieving 90% coverage of human papillomavirus (HPV) vaccination among girls as one of the core measures [1]. However, challenges related to the high vaccine procurement and delivery costs, logistical barriers, and supply constraints have led to ongoing slow uptake and low accessibility to HPV vaccines, particularly in low- and middle-income countries (LMICs) with a high burden of cervical cancer. The COVID-19 pandemic has further exacerbated financial, logistics, and supply constraints, resulting in only 12% HPV vaccine coverage for adolescent girls (9 − 14 years) in 2021 worldwide [2]. Accumulating evidence has indicated that one-dose vaccination schedules might provide comparable protection against persistent HPV infection as two-dose schedules [3–5]. The potential lower costs and simplified administration of the one-dose schedule have positioned it as a promising means to improve HPV vaccine uptake in populations with limited healthcare access. Clarifying the potential health and economic impacts of reduced-dose HPV vaccination programs can inform stakeholders in determining the optimal strategy for expanding HPV vaccination.

## Main text

In *BMC Medicine*, Prem and colleagues compared the long-term health benefits and cost-effectiveness of one-dose versus two-dose HPV vaccination in 188 countries, under scenarios in which the one-dose schedule provides either a shorter duration of protection or lower vaccine efficacy compared to two doses [6]. Prem et al.’s study revealed that if a single HPV vaccine dose confers ≥ 30 years of protection or lifelong protection but at 80% efficacy, the difference in population benefits of the one-dose versus two-dose vaccination schedule would be minimal [6]. Importantly, the study underscored that, in contrast to high-income countries (HICs), the one-dose schedule in LMICs would avert a greater number of cervical cancer cases and necessitate the vaccination of fewer girls per prevented case [6]. This highlights the value of the one-dose program for cervical cancer prevention in LMICs.

Inequality in the allocation of HPV vaccines persists globally, impeding the expansion of HPV vaccination initiatives. In recent years, ongoing shortages in vaccine supplies have disproportionately affected LMICs, leading to delays in their vaccination rollout [7]. In countries where HPV vaccination programs have been implemented, the average vaccine coverage in LMICs significantly lags behind that of HICs [8]. The recent COVID-19 pandemic has exacerbated inequality as the supply of HPV vaccines quickly declines in LMICs, while HICs have shown resilience [2]. A one-dose HPV vaccination program will significantly reduce program costs, simplify implementation, and ameliorate the challenges posed by vaccine supply shortages. Promoting widespread adoption of such simplified one-dose schedules has the potential to accelerate the introduction for countries that have yet to introduce the vaccine and to increase uptake in areas with low coverage, thereby narrowing the gap between HICs and LMICs. This shift could contribute significantly to the global goal of eliminating cervical cancer.

Prem et al. calculated the threshold cost of the second dose of the 9-valent HPV vaccine at which the two-dose schedule would remain cost-effective compared to a one-dose schedule. The study showed that the threshold cost for a second dose was only US$1.59 (median range: 0.14 − 3.82) in low-income countries (LICs) if one dose provides > 30 years of protection [6]. Remarkably, this cost threshold even falls below the lowest prices (US$4.50) established by the United Nations Children’s Fund Supply Division/Gavi [7]. This implies that the current minimum price of HPV vaccines may necessitate further downward adjustments to potentially render the two-dose vaccination program cost-effective in LICs. Despite the potentially shorter protection duration of the one-dose schedule compared to the two-dose schedule, the one-dose program emerges as a more cost-effective option for LICs. Introducing the one-dose program in these countries would prevent a larger number of eligible girls from missing vaccination. However, the initiation of a one-dose vaccination program also requires a significant upfront investment, potentially presenting barriers to introducing HPV vaccination in some countries. Further reductions in the price of HPV vaccines, especially the 9-valent vaccine, would also help LICs overcome significant financial barriers to introducing and sustaining HPV vaccine programs, thus maximizing the effectiveness of the one-dose schedule.

Achieving universal HPV vaccination necessitates concerted efforts from multiple stakeholders. Firstly, the augmentation of political will and leadership, along with the bolstering of interdepartmental coordination, remains paramount. Secondly, the implementation of educational initiatives targeting the general public and healthcare professionals holds promise in elevating the awareness and knowledge of HPV vaccines and overcoming vaccine hesitancy barriers. Thirdly, there persists a need for continued in-depth research to inform decision-making. The impact of the introduction of a one-dose HPV vaccination program on healthcare budgets is worth further investigation. For many countries, particularly those with limited financial resources, despite the well-defined cost-effectiveness of HPV vaccination, budgetary constraints and competing priorities within healthcare allocation might preclude affordability. Tailored to the specific contexts of these countries, the contemplation of gradual expansion of HPV vaccination coverage within budgetary feasibility stands as an avenue to supply valuable evidence to inform decision-making. A comprehensive investigation of the herd protection afforded by HPV vaccination and preventative effects against HPV-related diseases in both genders remains pertinent. This work will contribute significantly to deepening stakeholders' understanding of the full value of HPV vaccination and facilitating the introduction of HPV vaccination programs.

## Conclusions

The one-dose schedule emerges as a prospective strategy for scaling up HPV vaccination, particularly in LMICs. As data continue to emerge, continued exploration of potential differences in the effectiveness of one and two doses, as well as strategies to gradually expand HPV vaccination coverage within budgetary constraints, remains of paramount importance.

## Data Availability

Not applicable.

## Abbreviations

HIC: High-income country
HPV: Human papillomavirus
LIC: Low-income country
LMIC: Low- and middle-income country
